# RADx Variant Task Force Program for Assessing the Impact of Variants on SARS-CoV-2 Molecular and Antigen Tests

**DOI:** 10.1109/OJEMB.2021.3116490

**Published:** 2021-09-29

**Authors:** Richard S. Creager, John Blackwood, Thomas Pribyl, Leda C. Bassit, Anuradha Rao, Morgan Greenleaf, Filipp Frank, Wilbur Lam, Eric Ortlund, Raymond Schinazi, Alexander L. Greninger, Mia Cirrincione, Dale Gort, Emily B. Kennedy, Adam Samuta, Megan K. Shaw, Brian Walsh, Eric Lai

**Affiliations:** NaviDx Newport Beach CA 92660 USA; Biocomx Dana Point CA 92629 USA; Beck & Associates, Travelers Rest SC 29690 USA; Emory University 1371 Atlanta GA 30322 USA; Emory University 1371 Atlanta GA 30322 USA; Georgia Institute of Technology 1372 Atlanta GA 30332 USA; The University of Washington 7284 Seattle WA 98195 USA; Fred Hutchinson Cancer Research Center 7286 Seattle WA 98109 USA; ShoreFront Strategies Holland MI 49424 USA; OOMVELT Lakewood OH 44107 USA; Innovation Works Pittsburgh PA 15212 USA; Innova Group Alpharetta GA 30022 USA; Personalized Science San Diego CA 05403 USA

**Keywords:** COVID-19, in vitro diagnostics, mutations, SARS-CoV-2, variants of concern

## Abstract

*Goal:* Monitoring the genetic diversity and emerging mutations of SARS-CoV-2 is crucial for understanding the evolution of the virus and assuring the performance of diagnostic tests, vaccines, and therapies against COVID-19. SARS-CoV-2 is still adapting to humans and, as illustrated by B.1.1.7 (Alpha) and B.1.617.2 (Delta), lineage dynamics are fluid, and strain prevalence may change radically in a matter of months. The National Institutes of Health's Rapid Acceleration of Diagnostics (RADx^SM^) initiative created a Variant Task Force to assess the impact of emerging SARS-CoV-2 variants on *in vitro* diagnostic testing. Working in tandem with clinical laboratories, the FDA, and the CDC, the Variant Task Force uses both *in silico* modeling and *in vitro* testing to determine the effect of SARS-CoV-2 mutations on diagnostic molecular and antigen tests. Here, we offer an overview of the approach and activities of the RADx Variant Task Force to ensure test performance against emerging SARS-CoV-2 lineages.

## I. Introduction

On 31 December 2019, an emerging novel coronavirus was recognized and caused a public health emergency in Wuhan, China. The novel virus, previously called the 2019-novel coronavirus (2019-nCoV), is currently designated as the severe acute respiratory syndrome coronavirus-2 (SARS-CoV-2) [1]. As of 25 August 2021, this infection has been reported in 192 countries, causing over 210 million infections with 4.4 million deaths [2].

The first genetic sequence of SARS-CoV-2 (Wuhan-Hu-1) was made publicly available on 10 January 2020 [3]. Many *in vitro* diagnostic (IVD) nucleic acid amplification tests (NAAT) were developed using this sequence as a basis. In addition, antigens and antibodies were also derived from the virus with this sequence and used in the development of antigen assays for the direct detection of the virus.

Initial reports suggested that SARS-CoV-2 was not mutating rapidly, consistent with the slower evolutionary rates associated with coronaviruses [4]. Compared to human immunodeficiency virus, SARS-CoV-2 was changing much slower as it spread [5]. However, by April 2020, there were reports of a D614G mutation that was increasing in frequency at an alarming rate [6]. It is now clear that SARS-CoV-2 is accumulating mutations and diversifying into myriad lineages such as Alpha (B.1.1.7), Delta (B.1.617.2), and Gamma (P.1) variants [7].

Lineages such as Alpha and Delta do not represent homogeneous viral populations but instead comprise thousands of unique sequences. At the time of writing, there were more than 3.4 million sequences of SARS-CoV-2 in the database for the Global Initiative on Sharing Avian Flu Data (GISAID) [8] worldwide genomic database. SARS-CoV-2 genome sequencing in the United States has been greatly accelerated in 2021 by the Centers for Disease Control and Prevention (CDC) National SARS-CoV-2 Surveillance System (NS3) [9]; at present, there are more than 1,015,000 unique SARS-CoV-2 sequences in GISAID from the United States.

In February 2021, The United States Food and Drug Administration (FDA) issued the “Policy for Evaluating Impact of Viral Mutations on COVID-19 Tests: Guidance for Test Developers and Food and Drug Administration Staff**”** [10]. The FDA issued this guidance to provide a policy and recommendations on evaluating the potential impact of emerging and future viral mutations of SARS-CoV-2 on COVID-19 tests for the duration of the COVID-19 public health emergency. This guidance is applicable to NAAT and antigen tests that detect SARS-CoV-2 and to serology tests that detect antibodies to SARS-CoV-2.

The National Institute of Biomedical Imaging and Bioengineering (NIBIB) of the National Institutes of Health launched the Rapid Acceleration of Diagnostics (RADx^SM^) initiative in April 2020 to significantly increase the number, type, and availability of tests to diagnose the presence of SARS-CoV-2 [11]. In January 2021, NIBIB requested RADx form a task force dedicated to assessing the impact of SARS-CoV-2 variants on IVD testing. Most of the diagnostic tests on the market were developed based on the genome sequence (nucleotides and amino acids) of the Wuhan-Hu-1 strain of the virus. Clearly, mutations such as single nucleotide polymorphisms (SNPs), as well as insertions and deletions (indels), can negatively impact NAAT; also, amino acid substitutions, additions, and deletions can impact the target epitopes for immunoassays such as enzyme-linked immunoassays and lateral flow assays.

A team of scientists within RADx, Emory University, University of Washington, and across numerous United States government agencies quickly implemented the RADx Variant Task Force (RVTF), the activities of which currently comprise *in silico* genomic bioanalytical testing, sample collection, and clinical *in vitro* lab testing of technologies supported by the RADx program to ensure their detection efficacy with variants entering the population matches that of the original strain. This report summarizes the approach and methods of this program.

## II. RADx Variant Task Force (RVTF)

Shortly after the identification of the B.1.1.7 variant, the NIBIB called for the assembly of a task force to understand how the RADx portfolio of diagnostic tests are affected by emerging variants. The RVTF is a cross-disciplinary and cross-organizational group of scientists and industry leaders with expertise in virology and diagnostic testing.

The mission of the RVTF is to fill a gap between existing efforts, including the CDC NS3 program, FDA's oversight of IVD safety and accuracy, and the National Center for Biotechnology Information's maintenance of sequencing databases. While CDC is primarily focused on the analysis of surveillance data to anticipate and predict variants' effect on available vaccines and therapies, the RVTF is focused on how mutations found within variants affect the diagnostic performance of the RADx portfolio of IVD tests.

To meet the objective, the RVTF established:

1) strong and transparent collaborations with the CDC to obtain clinical specimens with complete amino acid and nucleotide sequences and the FDA for establishing robust experimental protocols;
2) *in silico* modeling for both NAAT and antigen tests to assess the potential impact of mutations on assay performance;
3) a comprehensive biobank containing multiple specimens for all known variants of concern (VOC) and variants of interest (VOI) in the United States;
4) laboratory test protocols for performing *in vitro* lab testing with VOC and VOI; and
5) the integration of lab results with bioinformatics and modeling to optimize and improve IVD tests.

## III. ROSALIND Diagnostic Monitoring (DxM)

**Fig. 1.**
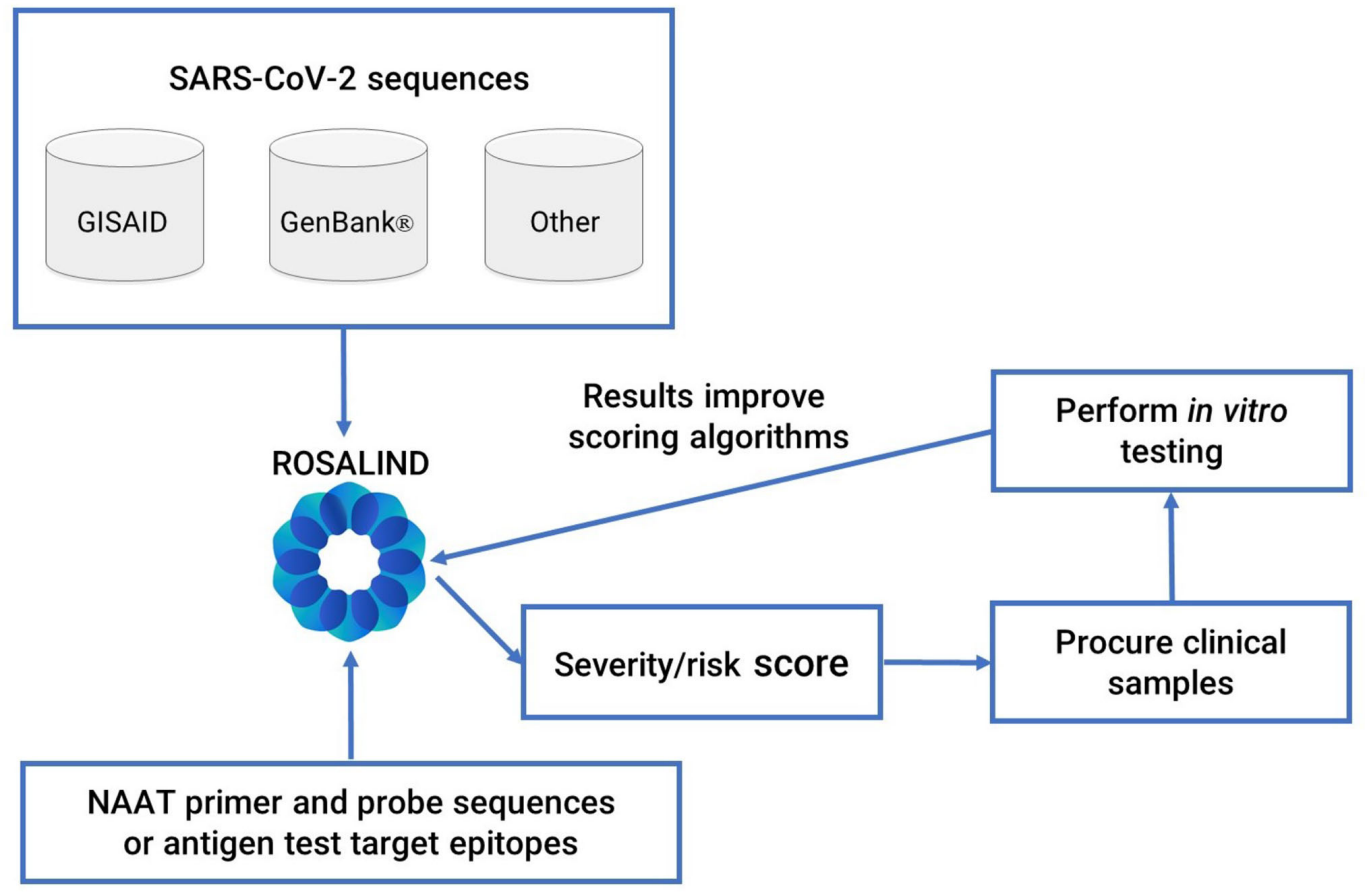
ROSALIND Diagnostic Monitoring (DxM) system. ROSALIND DxM automatically imports sequences from U.S. and global databases. NAAT developers upload primer and probe sequences, and antigen test developers upload target epitopes. ROSALIND DxM automatically assesses each test design against all available sequences and assigns a severity (NAAT) or risk score (antigen tests) based on the potential impact of emerging SARS-CoV-2 variants on diagnostic performance. Results of *in vitro* testing are fed back into ROSALIND to improve scoring algorithms.

In collaboration with ROSALIND (San Diego, California), the RVTF developed a comprehensive platform, ROSALIND Diagnostic Monitoring (DxM), that integrates bioinformatic modeling with clinical *in vitro* lab testing to assess the impact of SARS-CoV-2 mutations on both NAAT and antigen assays (Fig. 1).

**Fig. 2.**
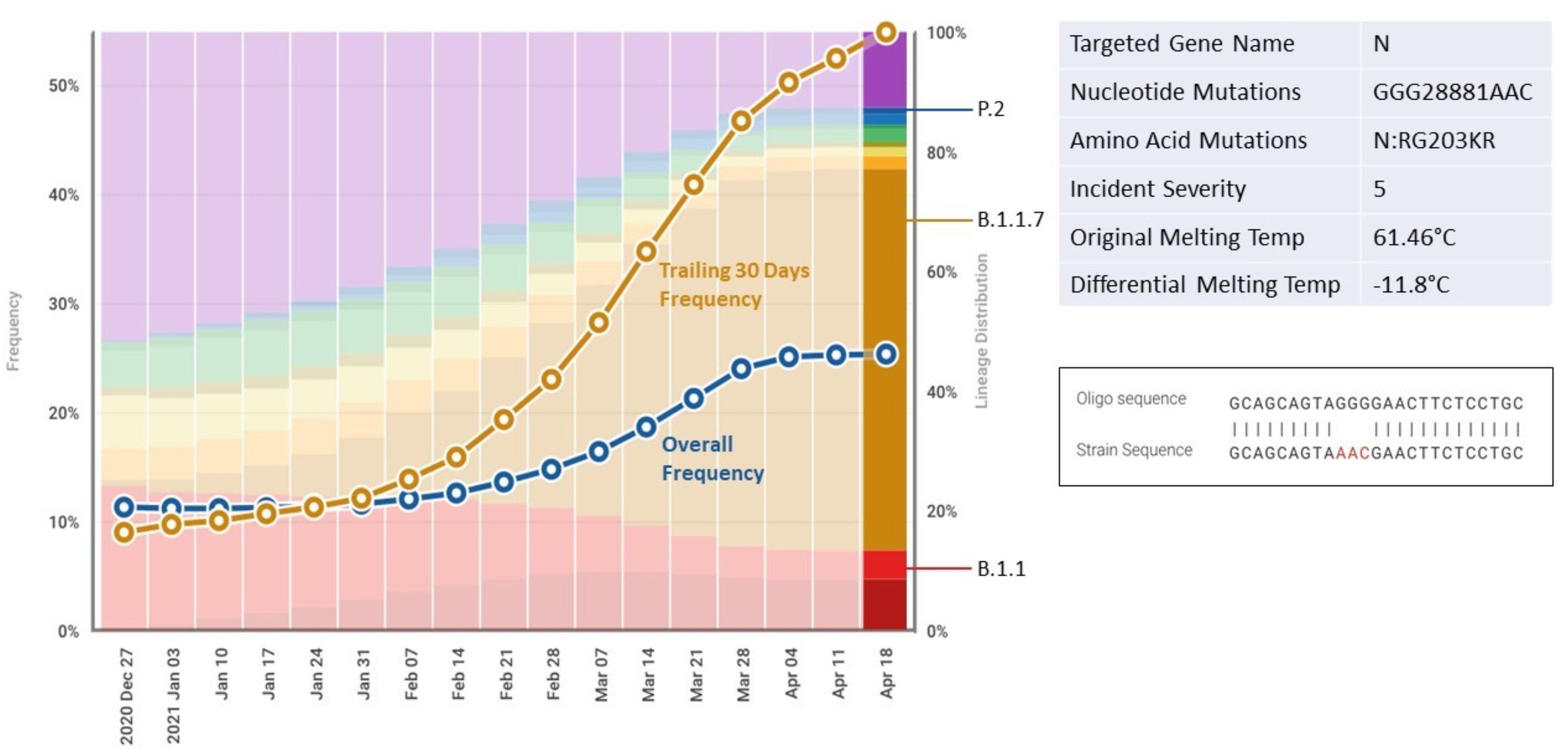
ROSALIND DxM incident detail. Example ROSALIND incident showing a mutation with a three base pair SNP in the middle of a primer resulting in a 11.8°C reduction in melting temperature and two amino acid changes.

DxM automatically pulls information from SARS-CoV-2 databases, such as GISAID and GenBank [12] open access genomic databases, as well as sequence information from clinical reference labs participating in the CDC NS3 program. The program employs the large-scale genome alignment tool (LASTZ) [13] to align each GISAID sequence to the Wuhan-Hu-1 strain (Wuhan/WIV04/2019). The software also uses the basic local alignment search tool (BLAST) [14] to find the position of primers and probes on the same Wuhan strain.

The software program allows each RADx test developer of a NAAT to enter their test's specific primer and probe sequences and automatically assess the test design against all sequences in the databases. The software also allows antigen test developers to enter their test's targeted epitope or protein region to assess the potential impact of amino acid changes on their assay. Nucleic acid and amino acid sequences are downloaded daily, which allows RADx test developers to perform their *in silico* analysis on demand.

### A. In Silico Analysis of NAAT Assays

DxM’ s severity rating for NAAT involves an assessment of the number and locations of SNP and indel mutations and the resulting change in temperature of melting (Tm) (Table I). The minimum severity score is zero (0) and the maximum severity score is five (5). The combination of incidents and severity scores is used to assess the overall potential impact to the performance of the assay [15].

**TABLE I:** NAAT Severity Score Calculation

Nucleotide Change	PCR	LAMP		FISH		CRISPR	
1 SNP	+ 0	+ 0		+ 1		+ 1	
2 SNPs	+ 1	+ 1		+ 2		+ 2	
3 SNPs	+ 2	+ 2		+ 3		+ 3	
>3 SNPs	+ 4	+ 4		+ 4		+ 4	
Adjacent SNPs	+ 2	+ 2		+ 2		+ 2	
1 base indel	+ 1	+ 1		+ 2		+ 2	
>2 bases indel	+ 3	+ 3		+ 3		+ 3	
SNP or Indel Location							
SNP in ultimate or penultimate base of 3' end of primer	+ 3/SNP	+ 3/SNP ^a^		N/A		N/A	
SNP in ultimate or penultimate base of 5' end of primer	N/A	+ 3/SNP ^b^		N/A		N/A	
SNP in 3rd, 4th, and 5th base from 5' end of primer	N/A	+ 3/SNP ^b^		N/A		N/A	
SNP in 3rd, 4th, and 5th base from 3' end of primer	+ 2/SNP	+ 2/SNP ^a^		N/A		N/A	
SNP in probe	+2/SNP	N/A		N/A		N/A	
Indel in last 5 bases of 3' end of a primer	+ 2	+ 2/SNP ^a^		N/A		N/A	
Indel in last 5 bases of 5' end of primer	N/A	+ 2/SNP ^b^		N/A		N/A	
Indel in probe	+ 2	N/A		N/A		N/A	
						
Delta Temperature of Melting (Tm)									
				
≥ 5 and < 8	+ 2	+ 2		+ 2		+ 2	
≥ 8	+ 4	+ 4		+ 4		+ 4	
				
				

^a^For F2,B2,F3,B3,FL,BL.

^b^For F1C,B1C.

### B. In Silico Analysis of Antigen Assays

An antigen test relies on the binding of a diagnostic “probe” (e.g., an antibody, nanobody, or aptamer) to an epitope within a viral protein (the antigen). Antigen mutations may affect the performance of the diagnostic test but predicting this is difficult due to the complex three-dimensional structure and the dynamic behavior of proteins in solution. This is further complicated by the fact that the location of the epitopes of most diagnostic probes are unknown.

The DxM software identifies amino acid changes and deletions and uses this information to generate risk scores. A simple scoring system was implemented that considers two factors: (1) the severity of the amino acid change and (2) the surface accessibility of the mutated residue. The severity of an amino acid change is based on general exchangeability scores derived from experimentally evaluated effects of mutations on protein function [16]. Surface accessibility scores were determined using published structures of spike and nucleocapsid proteins and calculations of the solvent accessible surface area for each residue [17]. For spike protein, both the open and closed conformations of the SARS-CoV-2 spike protein receptor binding domain were considered. Missing residues such as unresolved regions in the spike protein or regions predicted to be intrinsically disordered in the nucleocapsid protein were considered solvent accessible.

Recognizing the simplicity and low confidence of these scores and to improve reliability of the scoring system, the RVTF will perform experimental epitope mapping experiments. These include a structural approach using cryo-electron microscopy as well as a high-throughput assay involving a deep mutational scanning library of spike and nucleocapsid proteins to create a comprehensive approach to variant interpretation for a given diagnostic assay.

### C. Monitoring VOC/VOI

The DxM software provides RADx test developers an assessment of the frequency of specific SARS-CoV-2 mutations internationally, nationally, and regionally (e.g., by state). This information is available for the previous 30, 60, and 90 days from any analysis (Fig. 2).

The RVTF identifies VOC and VOI using the criteria established by the CDC and FDA:

1) Established VOC/VOI based on lineage and CDC definitions: e.g., Alpha (B.1.1.7), Beta (B.1.351), Delta (B.1.617.2), and Gamma (P.1 and P.2);
2) Variants based on mutations with potential biological implications: e.g., del69-70 (B.1.375); E484K (e.g., B.1.525 and B.1.526);
3) Variants or mutations that have reached a threshold of 5% prevalence in the United States or in a state; or
4) Variants that have a fast growth rate as determined by a prevalence doubling every 14 days nationally or regionally.

## IV. Clinical *In Vitro* Testing of VOC/VOI

The RVTF uses the VOC/VOI information to identify and obtain deidentified clinical samples to create a clinical repository (biobank) of SARS-CoV-2 variants in conjunction with the laboratories at Emory. The biobank will be used to create VOC/VOI panels for test developers to assess the performance of their tests on variants that were identified as potentially concerning by the *in silico* analysis. Each VOC/VOI panel will contain individual clinical samples that exhibit identical amino acid mutations and greater than 99.8% nucleotide homology. This testing will allow manufacturers to determine whether (1) their assay is able to detect specific variants, and/or (2) their assay's sensitivity is potentially impacted by a specific variant(s).

## V. Bioinformatic Modeling for Developing Improved Diagnostic Tests

DxM enables exploration of any mutations across all available international sequence data. Since January 2020, we have compiled, searched, and analyzed 3,459,536 (as of 25 September 2021) sequences in GISAID. This analysis showed that the S:D614G (S:A23403G) mutation, a non-synonymous mutation resulting in a replacement of aspartic acid with glycine at position 614 of the virus' spike protein, is present in almost all recent fast-spreading variants. This mutation in the viral spike protein occurred at the initial stage of the pandemic, emerging in late January to early February 2020; viruses containing glycine residue at position 614 became the dominant form of the virus globally, replacing the initial strain identified in China, by June 2020 [18], [19]. The S:D614G (S: A23403G) mutation is present in greater than 99% of sequences in the following lineages: B.1.1.7, B.1.427, B.1.429, B.1.525, B.1.526, B.1.617.2, C.37, and P.1. Of the 3.4 million sequences in GISAID, approximately 1% of samples spanning over 56 lineages do not contain the S:D614G mutation.

**Fig. 3.**
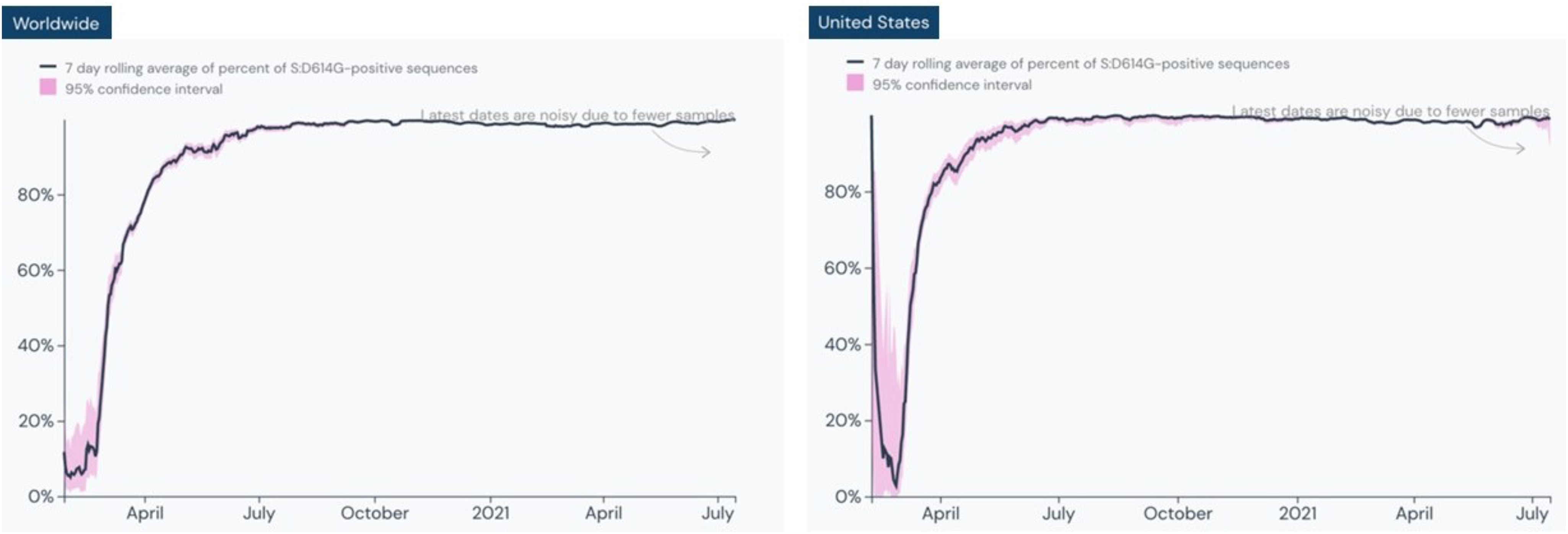
Seven-day rolling average percent of S:D614G-positive sequences worldwide (left) and in the United States (right). Image courtesy of outbreak.info [20].

Fig. 3 shows the prevalence of S:D614G globally and in the U.S. since the first quarter of 2020.

There is significant biological evidence as to why this mutation is important to the virus. The SARS-CoV-2 genome surrounding S:D614G is extremely stable. Structural and biochemical studies on a full-length G614 spike trimer showed that there are interactions absent in D614 that prevent premature loss of the S1 subunit that binds angiotensin-converting enzyme 2. This stabilization effectively increases the number of spikes that can facilitate infection [21].

## VI. Summary

The RVTF has established a comprehensive program that will enable RADx assay developers to comply with FDA recommendations for evaluating the impact of viral mutations on COVID-19 tests. The combination of the DxM software and the creation of the SARS-CoV-2 variant biobank provides a process by which RADx NAAT and antigen test developers will be able to design their tests to minimize the impact of viral mutations on test performance and routinely monitor for viral mutations that may impact test performance.

To date, the RVTF contracted with three clinical reference labs participating with the CDC NS3 national surveillance project. The biobank contains more than 85,000 clinical specimens, some of which are heat inactivated and some of which are not inactivated. The biobank includes most VOC and VOI that have circulated in the United States since mid-2020. More than 60 assays are undergoing clinical *in vitro* testing including more than 15 which were granted FDA Emergency Use Authorization (EUA) status and approximately 45 others that are in development. The data for the FDA EUA tests will be published upon completion of the studies.

Bioinformatic modeling has revealed targets that should allow the development of improved diagnostic assays that can detect all SARS-CoV-2 variants. Additional studies are underway to identify other variant agnostic targets as well as potential SNPs that can be used to differentiate variants. The extensive clinical sample biobank will support the rapid validation of these new tests.
